# Draft Genome Sequence of “Nocardia suismassiliense” Strain S-137 (CSUR P4007)

**DOI:** 10.1128/genomeA.00212-18

**Published:** 2018-04-19

**Authors:** Mustapha Fellag, Anthony Levasseur, Jérémy Delerce, Fadi Bittar, Jean-Lou Marié, Bernard Davoust, Michel Drancourt

**Affiliations:** a Aix-Marseille Université, MEPHI, IRD, IHU-Méditerranée Infection, Marseille, France; b Animal Epidemiology Working Group of the Military Health Service, DRSSA, Toulon, France

## Abstract

“Nocardia suismassiliense” strain S-137 isolated from Sus scrofa feces exhibits a 9.4-Mb (67.1% GC content) draft genome sequence containing 8,658 protein-coding genes, 66 tRNAs, and 9 rRNAs. *In silico* DNA-DNA hybridization confirmed strain S-137 as representative of a new species, “Nocardia suismassiliense,” closely related to N. tenerifensis and N. brasiliensis.

## GENOME ANNOUNCEMENT

The genus Nocardia, named in honor of the French veterinarian Edmond Nocard (1), contains 115 species in the List of Prokaryotic Names with Standing in Nomenclature (http://www.bacterio.net/nocardia.html). Belonging to a suborder of aerobic actinomycetes, the genus Nocardia comprises opportunistic pathogens that cause localized and disseminated infections in humans and in animals (2). However, there is little information about diversity and pathogenicity of Nocardia species in animals, and only one report has been issued for wild boars (Sus scrofa) (3). “Nocardia suismassiliense” strain S-137 (CSUR P4007) was isolated from the feces of a wild boar in the military camp at Canjuers, southeastern France (43°38′49″N; 06°27′56″E), using chlorhexidine decontamination and culture on MOD9 medium (4). We analyzed its whole-genome sequence in order to describe its genomic content and help develop molecular identification tools.

Nocardia sp. strain S-137 was subcultured on Colombia agar incorporating 5% sheep’s blood (bioMérieux, Marcy l’Etoile, France) at 37°C. DNA extracted using an EZ1 biorobot and an EZ1 DNA tissue kit (Qiagen, Courtaboeuf, France) for <50 µL volume, was quantified by a Qubit assay with a high-sensitivity kit (Life Technologies, Carlsbad, CA, USA) to 23.1 ng/µL. DNA was then sequenced using MiSeq technology (Illumina, Inc., San Diego, CA, USA) with paired-end and mate pair applications. The index representation for strain S-137 was 6.98%. A total of 866,229 paired-end reads were filtered per read qualities and assembled using SPAdes software (5). Contigs were combined by using SSPACE (6), GapFiller (7), and manual finishing using similarity searches and synteny block detection.

The strain S-137 draft genome was assembled into 25 scaffolds composed of 142 contigs, with a total size of 9,465,473 bp and a GC content of 67.1%. Automatic annotation was performed with Prokka version 1.12 (8). The genome presents two repeat regions and is predicted to encode 8,658 genes, including 8,582 protein-coding genes and 76 RNA genes, including 66 tRNAs, 1 transfer-messenger RNA, 3 5S rRNAs, 3 23S rRNAs, and 3 16S rRNAs. The three 16S rRNA gene copies exhibit a total of 17 nucleotide differences and were compared with the reference N. brasiliensis genome (GenBank accession number CP022088), resulting in a similarity score of 98% for one copy, while the two other copies match with 99% similarity.

Based on the 16S rRNA gene sequence proximity, genomes were selected and incorporated into *in silico* DNA-DNA hybridization (DDH) (9). The DDH values were calculated using the Genome-to-Genome Distance Calculator (GGDC) version 2.0 online tool (10). This analysis yielded 31.2% sequence similarity with the type strain N. tenerifensis NBRC 101015 (NCBI reference sequence NZ_BAGH00000000), 27.5% sequence similarity with N. brasiliensis ATCC 700358 (NC_018681), 24.9% sequence similarity with N. asiatica NBRC 100129 (NZ_BAFS00000000), 24.8% sequence similarity with N. abscessus NBRC 100374 (NZ_BAFP00000000), and 24.5% sequence similarity with N. arthritidis NBRC 100137 (NZ_BDBB00000000). These data indicate that strain S-137 is representative of a new species, “Nocardia suismassiliense,” that is closely related to N. tenerifensis and N. brasiliensis.

These data illustrate the importance of whole-genome sequencing in the taxonomic study and identification of new Nocardia species, since 16S rRNA gene sequencing may be insufficient for distinguishing closely related species.

### Accession number(s).

The genome sequence reported here has been deposited at EMBL/GenBank under the accession number OIFR00000000. The version described here is the first version, OIFR01000000.
